# Unraveling the drivers of regional variation in healthcare spending by analyzing prevalent chronic diseases

**DOI:** 10.1186/s12913-018-3128-4

**Published:** 2018-05-03

**Authors:** Eline F. de Vries, Richard Heijink, Jeroen N. Struijs, Caroline A. Baan

**Affiliations:** 1 Tilburg University, Tranzo, Tilburg School of Social and Behavioral Sciences, PO Box 90153, 5000 LE Tilburg, The Netherlands; 2 Department of Quality of Care and Health Economics, National Institute of Public Health and the Environment (RIVM), Center for Nutrition, Prevention and Health Services, PO Box 1, 3720 MA Bilthoven, The Netherlands; 3 Dutch Healthcare Authority, PO Box 3017, 3502 GA Utrecht, The Netherlands

**Keywords:** Regional variation, Healthcare spending, Lmm, Disease-approach

## Abstract

**Background:**

To indicate inefficiencies in health systems, previous studies examined regional variation in healthcare spending by analyzing the entire population. As a result, population heterogeneity is taken into account to a limited extent only. Furthermore, it clouds a detailed interpretation which could be used to inform regional budget allocation decisions to improve quality of care of one chronic disease over another. Therefore, we aimed to gain insight into the drivers of regional variation in healthcare spending by studying prevalent chronic diseases.

**Methods:**

We used 2012 secondary health survey data linked with claims data, healthcare supply data and demographics at the individual level for 18 Dutch regions. We studied patients with diabetes (*n* = 10,767) and depression (*n* = 3,735), in addition to the general population (*n* = 44,694). For all samples, we estimated the cross-sectional relationship between spending, supply and demand variables and region effects using linear mixed models.

**Results:**

Regions with above (below) average spending for the general population mostly showed above (below) average spending for diabetes and depression as well. Less than 1% of the a-priori total variation in spending was attributed to the regions. For all samples, we found that individual-level demand variables explained 62-63% of the total variance. Self-reported health status was the most prominent predictor (28%) of healthcare spending. Supply variables also explained, although a small part, of regional variation in spending in the general population and depression. Demand variables explained nearly 100% of regional variation in spending for depression and 88% for diabetes, leaving 12% of the regional variation left unexplained indicating differences between regions due to inefficiencies.

**Conclusions:**

The extent to which regional variation in healthcare spending can be considered as inefficiency may differ between regions and disease-groups. Therefore, analyzing chronic diseases, in addition to the traditional approach where the general population is studied, provides more insight into the causes of regional variation in healthcare spending, and identifies potential areas for efficiency improvement and budget allocation decisions.

**Electronic supplementary material:**

The online version of this article (10.1186/s12913-018-3128-4) contains supplementary material, which is available to authorized users.

## Background

The sustainability of healthcare systems is a common challenge for Western countries. One of the main responses is to improve population health and quality of care, while slowing down the expenditure growth by population health management (PHM). PHM aims to integrate services across healthcare, prevention, social care, welfare and public health for a pre-specified population within the region [1]. Commonly, these initiatives are set-up by a network of health insurers, health care providers and other health organizations that together develop interventions. These interventions include so-called citizen-centered interventions, which targets citizens at risk and are tailored for pre-defined subpopulations such as citizens who smoke, diabetes patients or multi-morbid patients. In a number of countries, stakeholders within specific regions have adopted the PHM approach. Examples are Gesundes Kinzigtal in Germany [2], Accountable Health Communities in the USA [3] and Dutch PHM initiatives [4, 5]. In order to develop policy to improve efficiency and to make budget allocation decisions, these initiatives require insight into the performance of (the organization of) their health services. One way to inform these decisions is to study the drivers of variation in healthcare spending between regions, as regional variation in healthcare spending, not caused by differences in medical need, is said to *indicate* inefficiency [6, 7].

Research has shown that regional variation in healthcare spending is determined by the interplay of demand and supply factors under the influence of system factors [8, 9]. Demand factors labeled as medical need, such as demographics and health status, are generally considered justifiable causes of variation in healthcare spending. Empirical research has shown that such factors explain a large part of the regional variation in healthcare spending (e.g. [8, 10, 11]). Regional variation as a result of supply factors (e.g. competition, capacity or physician beliefs) is considered to be generally undesirable. In empirical studies, those factors have been found to be of varying impact, depending on the study context, or system factors (e.g. [11, 12]). System factors such as (insurance) regulation, price setting or payment methods influence the dynamics of demand and supply.

Previous studies (e.g. [6, 10, 13]) generally examined regional variation in healthcare spending studying sources of variation that affect spending for the entire population in a region, such as local price levels or general economic circumstances. However, there is substantial population heterogeneity that clouds a more detailed interpretation, as other causes of variation might differ between disease groups across regions. Population heterogeneity between regions exists, for example in terms of the prevalence of (chronic) diseases and multi-morbidities; a region may have a relatively high prevalence for certain disease groups and lower prevalence for others. Furthermore, the extent to which regional differences are caused by disease severity or other patient characteristics or the level of treatment standardization may vary between disease groups [14, 15]. These more detailed explanations remain undetected when studying the general population only. Also, because of lack of knowledge and data, it is complicated to control for all relevant sources of variation in healthcare spending. In order to inform, for instance, budget allocation decisions for quality improvements or care standardization efforts of one chronic disease over another, a more detailed approach may be needed.

Therefore, this study aimed to gain further insight into the sources of regional variation in healthcare spending by zooming in on prevalent chronic diseases (disease-approach). We applied this to the context of 18 Dutch PHM regions. We expected variation patterns to differ between disease-groups within the population. This is in part intrinsically to the disease and in part in line with the degree to which consensus is reached on how to treat a certain disease [8, 15]. We selected two types of prevalent disease groups: patients with diabetes and patients with depression. We chose patients with diabetes as treatments for diabetes are known to be highly standardized due to provider approved general treatment decisions in the Dutch Diabetes Federation Health Care Standard (DFHCS) and as a consequence of the bundled payment model that was introduced in 2010 [16]. We chose patients with depression as treatments for depression are expected to be less standardized, considering the variety of treatment options and lack of healthcare standards at a national level. Assuming that treatment options vary in costs, we expected the group of patients with depression to reflect more variation in healthcare spending as compared to the group of patients with diabetes. We showed the results of the disease-approach in addition to the results of the traditional approach (where the general population is studied) for each step taken in the analysis of regional variation in spending, which is to 1) describe the unadjusted regional variation in healthcare spending, and 2) explore the extent to which demand and supply factors explain regional variation in healthcare spending.

## Methods

### Data sources

We used data from the Dutch Public Health Monitor (DPHM) for the year 2012 [17]. We merged these data at the individual level with demographic variables from Statistics Netherlands and nationwide claims data which we obtained from Vektis [18]. The claims data included all health care use in 2012 that was covered by the basic health insurance. The records were linked using a pseudonymized personal identification code. Furthermore, at the regional level, we added health care supply data from the Netherlands Institute for Health Services Research (NIVEL), the National Institute of Public Health and the Environment (RIVM) and the Health Care Inspectorate (IGZ). We used 2012 as reference year for all data sources. For an overview of data and variables used, see Additional file 1.

### Study population

To define the regions, we used the geographical demarcation of 18 Dutch PHM initiatives [4]. We analyzed the variation in healthcare spending across these regions, focusing on three (sub)populations: 1) the general population, 2) patients with diabetes, and 3) patients with depression. For a detailed description of the sample selection, see Additional file 2.

The first sample (general population) was based on participation in the DPHM survey (*n* = 138,000), since we aimed to include health status information from that survey. We excluded individuals with missings on self-reported health status (*n* = 82,455) and missings on the health spending variable (*n* = 10,298). In addition, to correct for errors and costs in the data that are not linked to Diagnosis Treatment Groups (DTGs) in the previous year, we removed 1% outliers in terms of spending (above €29,468) (*n* = 1,284) and negative values for health spending (n = 1). As a result, 44,694 individuals from the general population were included in the sample. The second study sample consisted of diabetes patients. We selected them by their participation in a bundled payment program for diabetes care, the Pharmacy-based Cost Group (PCG) or the DTGs referring to diabetes type II (*n* = 10,767), which is known to include nearly all diabetes type II patients (Elissen A, BiloH, Struijs J, Van Galen M, De Vries R, Luijk R, et al. Development of a national diabetes register based on claims data to support population health management in the Netherlands, under review). The PCGs are – among other variables - used in the Dutch risk equalization system to identify risk-profiles that predict healthcare spending in the following year [19]. PCGs aim to identify persons with chronic diseases based on claims data of the previous year for medication for which it is known that they are used for that particular disease. The third study sample was created by selecting patients with depression (*n* = 3,735) in a similar way as the diabetes patients. We used PCGs and DTGs referring to depression.

### Econometric specification

To allow for the complex structure of the data, i.e. individuals nested within regions and the skewed distribution of healthcare costs as dependent variable, we performed multilevel analyses using linear mixed models (LMM) with a random intercept for region effects. We applied LMM to the log-transformed dependent variable. We considered other methods and distributions: see Additional file 3 for details on the model selection. We specified the following eq. (1):

1 $$ \log \left({y}_{ij}\right)={\beta}_0+\sum {\beta}_{ij}\ {X}_{ij}+\sum {\beta}_{0j}\ {X}_{0j}+{\nu}_{0j}+{\varepsilon}_{ij} $$

where *y*_*ij*_ is the healthcare spending of individual i in region j, *β*_*ij*_ are the fixed effects of individual level characteristics; *X*_*ij*_ is the vector of variables at the individual level; *β*_0*j*_ are the fixed effects of the region level characteristics; *X*_0*j*_ is the vector of variables at the region level; *ν*_0*j*_ is the random intercept at the region level with *ν*_0*j*_~*N*(0, *σ*_*v*_^2^), and *ε*_*ij*_ is the residual error at the individual level with *ε*_*ij*_~*N*(0, *σ*_*ε*_^2^).

The outcome variable was the natural log of total curative healthcare spending in 2012 at the individual level (see Additional file 4). It was calculated by summing spending on general practitioner (GP)-care, hospital and specialist care, pharmaceutical care, physical therapy, mental health care and other types of care (i.e. patient transport, maternity care, medical aids and care abroad). It comprised all spending within the basic health insurance package [18].

Similar to previous studies, individual characteristics (age, gender, SES and health status) were included in the model. These variables reflect justified causes of variation in healthcare spending, which is also referred to as medical need (e.g. [6]). The self-reported health status variable was derived from the DPHM survey consisting of three levels from bad to very good [17]. Self-reported health status is generally considered to be a good predictor for future health as it encompasses more than standard objective measure of health (e.g. blood pressure, presence of disease) and reflects preclinical diseases or worsening of diseases [20]. In addition, using the self-reported health status in addition to using claims data derived health status alone, resolves the bias resulting from claims data derived health status, which is not independent from the supply-side and therefore may also reflect supplier-induced utilization. We used 2008 and 2012 Diagnosis-based Cost Groups (DCGs), which are cost-profiles of the diseases based on diagnosis information [21] and PCGs from the Dutch risk equalization model [19], each referring to data from previous years. Using claims data from previous years may tackle the endogeneity issues that arise when using spending and health data from the same year [22]. The DCG variable ranged from 0 to 13 or 15 (13 clusters for 2008 and 15 for 2012) where a higher DCG number is equal to being in a higher cost cluster. We transformed the DCG into dummy variables, reflecting being in either a specific DCG or not. The PCG variable ranged from 0 to 20 or 25 (20 clusters for 2008 and 25 for 2012), where a higher number means being in a higher cost cluster. As it was possible for an individual to be in more PCGs at the same time (i.e. reflecting multiple diseases), we constructed an aggregate score from the PCG by summing the PCGs for each individual.

Supply variables, in this study mainly about the access to healthcare, reflecting generally unjustified variation, were included at the individual and at the regional level. At the individual level, we added distance to provider in meters from Statistics Netherlands. At the regional level, we used number of providers per 1000 population, which we constructed using the four-digit postal codes of providers in the Netherlands using information from NIVEL, RIVM and IGZ.

The random intercept at the regional level in the full model is considered to *indicate* variation in efficiency between regions, as it reflects regional variation which is not due to the demand and supply variables and the random error at the individual level in the model.

### Statistical analyses

First, in order to gain insight in the a-priori variation of the three samples, we showed the deviation from the mean in percentages per region and the overall coefficient of variation (CoV; ratio of the standard deviation and the mean) at the regional level. The a-priori regional variation includes all justified and unjustified variation in healthcare spending between regions.

Second, we studied the extent to which demand and supply factors explained variation in healthcare spending at the individual and regional level. For all samples separately, to identify the contribution of the variables to the model, we fitted LMM models by gradually adding groups of variables to a null model without covariates. This strategy is common in analyzing regional variation in healthcare spending and is previously used by e.g. Gopffarth et al. [11] and Newhouse et al. [6]. We started with demographics (age and gender), followed by health status variables (first self-reported health status and then the DCGs and PCGs). Consecutively, we added the supply variables one by one. Finally, we selected the best-fit model based on subsequent nested fits for each sample using the likelihood ratio test (LRT), Akaike Information Criteria (AIC) and Bayesian Information Criterion (BIC). We chose the more expanded model when the *p*-value for the LRT < 0.01, and checked whether the AIC and BIC corresponded accordingly. After fitting the model, we calculated Pearson’s correlation coefficients for the observed and predicted means of natural log of the curative healthcare spending to estimate how the predicted means changed by adding the covariates. In addition, we plotted the variance at each level for all samples.

Data management and analyses were performed using SPSS Version 20 and Stata/MP 14.0.

## Results

### Unadjusted regional variation in healthcare spending

**Fig. 1:**
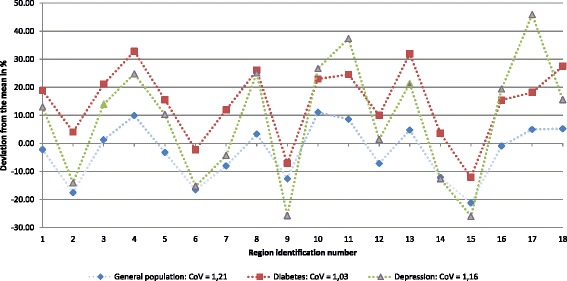
Unadjusted regional variation in healthcare spending in the general population and the disease-approach. *CoV: Coefficient of Variation (ratio of standard deviation and mean); data label: region identification number*

Figure 1 displays the unadjusted regional variation shown as deviation from the mean in percentages per sample per region. It includes both justified and unjustified variation in healthcare spending. For the diabetes sample, region 13 and 4 ranked highest in spending per capita (pc). The two lowest spending regions were 15 and 9. The depression sample had highest spending pc in regions 17 and 11; lowest in region 15 and 9 (see Additional file 4 for more details and for the exact levels of spending per region). Regions with above (below) average spending for the general population mostly showed above (below) average spending for diabetes and depression as well. The disease-groups showed less variability compared to the general population (CoV of 1.21). The diabetes sample showed less variation than the depression sample, which is reflected in the CoV of 1.03 for diabetes and 1.16 for depression.

### Variance (un)explained by demand and supply factors

Table 1 shows the model estimates for the null and the best-fitted model for all samples. The results are shown on the log-transformed scale. For all samples, the best-fitted model included demand variables as demographic variables and health status variables (both self-reported and claims data derived). Additional file 5 illustrates that self-reported health status explains a substantial part of healthcare spending; the total variance decreased approximately 28% on the log-scale. Supply variables were found to add small but significantly on the log-scale, to the models for depression and the general population (not for diabetes). The best-fitted depression model included distance in meters to a physical therapist. For the general population, distance in meters to GP, hospital and pharmacy were additionally included. The Pearson’s correlations (Table 1) between the overall mean in the null models and predicted mean in the full models confirmed the influence of the covariates by showing coefficients of 0.12 (diabetes), 0.14 (general population) and 0.16 (depression), which was largely due to the influence of the demand variables.

**Table 1:** LMM model estimates for the traditional approach and disease-based approach

				General Dutch population (*n* = 44,694)		Diabetes (*n* = 10,767)		Depression (*n* = 3,735)	
				Model 0	Model 10	Model 0	Model 5	Model 0	Model 9
	variable			Beta (se)	Beta (se)	Beta (se)	Beta (se)	Beta (se)	Beta (se)
		intercept (patient level)		7,64 (0,03)***	6,21 (0,03)***	8,04 (0,02)***	6,95 (0,05)***	7,72 (0,05)***	6,20 (0,08)***
demand		age			0,01 (0,00)***		0,00 (0,00)***		0,01 (0,00)***
		gender			0,03 (0,01)***		0,04 (0,01)***		0,03 (0,03)
	self-reported health status	fair			0,44 (0,01)***		0,31 (0,01)***		0,60 (0,04)***
		poor			0,77 (0,02)***		0,59 (0,02)***		0,94 (0,05)***
	claims data derived	DCG 2012	1		0,90 (0,03)***		0,68 (0,03)***		0,80 (0,14)***
			2		0,79 (0,02)***		0,54 (0,04)***		0,75 (0,08)***
			3		0,81 (0,02)***		0,60 (0,04)***		0,78 (0,10)***
			4		1,02 (0,02)***		0,81 (0,03)***		1,05 (0,07)***
			5		1,09 (0,02)***		0,86 (0,03)***		1,02 (0,09)***
			6		1,04 (0,02)***		0,92 (0,03)***		0,97 (0,08)***
			7		1,13 (0,03)***		0,99 (0,04)***		1,03 (0,13)***
			8		1,30 (0,06)***		1,06 (0,11)***		1,37 (0,21)***
			9		1,25 (0,04)***		1,08 (0,06)***		0,86 (0,18)***
			10		1,38 (0,04)***		1,18 (0,07)***		1,12 (0,17)***
			11		1,45 (0,12)***		1,35 (0,17)***		1,07 (0,48)**
			12		1,14 (0,06)***		1,04 (0,09)***		1,10 (0,29)***
			13		1,83 (0,09)***		1,68 (0,14)***		1,55 (0,32)***
			14		1,96 (0,31)***		1,49 (0,69)**		(omitted)
			15		1,99 (0,50)***		(omitted)		2,34 (0,96)**
		PCG 2012			0,250 (0,01)***		0,19 (0,01)***		0,22 (0,02)***
supply	distance to care provider in meters								
		GP			0,00 (0,00)				
		pharmacy			0,00 (0,00)				
		hospital			0,00 (0,00)				
		physical therapist			0,00 (0,00)***				0,00 (0,00)***
	intercept – variance at the regional level			0,02 (0,01)	0,00 (0,00)	0,01 (0,00)	0,00 (0,00)	0,04 (0,02)	0,00 (0,00)
	random error – variance at the individual level			1,24 (0,01)	0,75 (0,01)	0,79 (0,01)	0,48 (0,01)	1,49 (0,03)	0,92 (0,02)
Postestimation statistics	ICC			0,02 (0,01)	0,00 (0,00)	0,01 (0,00)	0,00 (0,00)	0,02 (0,01)	0,00 (0,00)
	AIC			136507	114297	28054	22696	12120	10351
	BIC			136533	114532	28075	22856	12139	10494
	log likelihood			−68250	− 57122	−14024	−11326	− 6057	− 5153
	PC mean and predicted mean				0,14***		0,12***		0,16***
	PC empirical Bayes mean				0,90***		0,94***		0,70***
	total variance on the log-scale			1,54	0,57	0,62	0,23	2,22	0,85
	total variance individual level			1,54	0,57	0,62	0,23	2,22	0,85
	total variance regional level			0,00	0,00	0,00	0,00	0,00	0,00

*DCG* diagnosis cost group, *PCG* Pharmacy-based cost group, *GP* General Practitioner, *AIC* Aikaike Information Criterium, *BIC* Bayesian Information Criterion, *PC* Pearson’s correlation; **: p-value < 0.05; ***: p-value < 0.01; *re* random effects

**Fig. 2:**
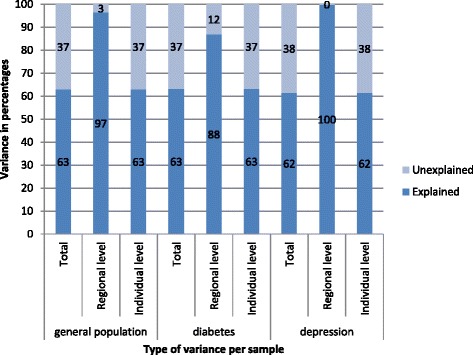
Variance in healthcare spending explained by covariates in the general population and the disease-approach

Figure 2 demonstrates the proportion of the variance that is explained after fitting the models. A-priori, more than 99% of the total variance was attributed to the individual level (also see Table 1), leaving less than 1% attributed to the regional level. At the individual level, 62-63% of the variance on the log-scale was explained by demand variables. At the regional level, the covariates explained relatively more variation for the general population and the depression sample (96% and 100% respectively) than for the diabetes sample (88%).

## Discussion

The aim of this study was to gain more in-depth insight into regional variation in healthcare spending using prevalent chronic diseases. We used samples of patients with diabetes and depression, as we expected more homogeneity due to treatment standardization in the first compared to the latter. To our knowledge, we were the first to apply such an approach. We found indications that levels and sources of variation in healthcare spending seem to differ between disease-groups. The results showed that unadjusted regional variation in healthcare spending was smaller for the sample of patients with diabetes, than for patients with depression and the general population. Regions with above (below) average spending for the general population mostly showed above (below) average spending for diabetes and depression as well. A-priori, more than 99% of the total variance was concentrated at the individual level, leaving less than 1% at the regional level. We found that demand variables explained 62-63% of the total variance in healthcare spending. Self-reported health status was the most prominent variable in the model, explaining 28% of the variation. Supply variables added nearly 0%, but significantly to the model in the general population sample (distance to GP, hospital, pharmacy and physical therapy) and the depression sample (distance to physical therapy). For the general population and the depression sample, the covariates in the model explained 96-100% of the variation at the regional level as opposed to 88% for the diabetes sample.

The finding that variation in healthcare spending is largely explained by demand factors and that the variation at the regional level is limited, is in accordance with work from e.g. Lavergne, Barer [23] and Gopffarth, Kopetsch [11]. Both studies were performed using a traditional population-based approach and performed their studies in similar health systems as the Netherlands. Based on their results, both authors concluded that regional differences in healthcare spending do not clearly reflect inefficiencies. Therefore, Lavergne, Barer [23] suggests that policy reforms should be rather targeted at system wide efficiency improvements, than at high-spending regions. Although the results of our study similarly showed a small amount of variation that was left at the regional level, we think that unexplained variance that might indicate inefficiency cannot be ruled out based on two findings. First, at the regional level, the diabetes sample showed relatively more unexplained variance than the other samples. This indicates that variance at the regional level was caused by factors outside of the model, as for example differences in efficiency in organizations that were influenced by the disease standardization. In contrast, the other study samples showed less variability. For the depression sample, all of the variation was explained at the regional level. This might be caused by variability in disease severity or treatment differences between individuals within regions, rather than across regions. Second, at the individual level, supply variables were small, but significant in the general population and depression sample and not in the diabetes sample. Even though we found small regional effects, we believe this insight is useful, as more efforts at the regional level are expected in the near future, and therefore more variability at the regional level may be encountered in analyses of disease-groups.

Our study has several limitations. First and most important, by using the self-reported health status variable which we derived from the Dutch Public Health Monitor survey, we were limited to a subsample of the nationwide patient-level claims data. Consequently, we encountered loss of precision in our analyses as the region sample sizes were less than 30 for a maximum of three regions in the disease-groups. Additionally, the external validity of this study depends directly on that of the Dutch Public Health Monitor, which shows selection bias that was corrected for by using a weighing factor (unpublished work of Carolien vd Brink submitted to TSG). Nevertheless, we believe that for the purpose of this study, namely to show a method on how to gain insight into differences between disease-groups within and across regions, this had no substantial impact on our conclusions. Second, controlling for supply and demand factors in the analyses should be improved. For example, we were not able to capture variables that inform cultural differences between physician treatments or the level of standardization of treatments per region. Third, due to computational issues in STATA we could not retransform the log-transformed healthcare spending variable and calculate variance measures on the linear scale (see Additional file 3). Interpretation of the results is therefore more difficult and less precise. Consequently, the extent to which variance is explained might differ when measured on the linear scale. However, and in extension to previous work [11, 24], we used a more complex hierarchical structure to account for the nested structure of individuals into regions and therefore avoided the disadvantages (i.e. ecological fallacy) of an aggregate design. As this study has a descriptive character and we did not aim to infer causally, we are confident our conclusions hold up in a qualitative sense.

Despite these limitations, this study contributes to the literature that aims to investigate the role of demand and supply factors in explaining geographical variation in health care spending. First, this study benefits from the homogenous Dutch healthcare system. In contrast to, for example, the US, financing structures and schemes are defined at the country level in the Netherlands. Therefore, they do not influence regional variation in healthcare spending. In addition, as opposed to studies from the U.S., Dutch claims data cover roughly 95% of the population, as private health insurance is mandatory. Second, we were able to reduce bias that results from using claims data derived health status, which was encountered in e.g. Lavergne, Barer [23] and Wennberg, Sharp [25], by including a self-reported health status variable from a large health survey. Finally, by using a disease-approach, we were able to create a more homogenous study population in advance, which enriches the understanding of causes of regional variation in spending between groups within the population. To our knowledge, we are the first to apply such an approach. It provides a novel process of modeling regional variation in healthcare spending that may be followed in similar future studies. The notion that regional variation in healthcare spending might be a composition of different variation patterns of several disease-groups is important when developing regional healthcare policies. We suggest further research to investigate whether regions are consistent over a larger set of disease-groups and specific cost data to better interpret the results research has shown this far. Moreover, in the coming years data will become available to analyze the effects of the PHM regions. Additionally, more research is needed to fill in the gaps of regional variation that remains unexplained. A suggestion is to operationalize cultural differences to include in the analysis (previously mentioned by Kopetsch and Schmitz [24]). At the individual level, cultural differences might influence healthcare utilization on the demand side and at the organizational level or physician level cultural differences may influence the supply side. In addition, a longitudinal analysis of regional variation in healthcare spending using disease-groups might be interesting to start unraveling the causal relationship instead of a more descriptive analysis as used in this study.

## Conclusion

The extent to which regional variation in healthcare spending can be considered as inefficiency may differ between regions and disease-groups. Therefore, an approach analyzing chronic diseases, in addition to the traditional approach, where the general population is studied, provides more detailed insight into the causes of regional variation in healthcare spending and identifies potential areas for efficiency improvement and budget allocation decisions.

## Additional files

Additional file 1: Data and sources. (DOCX 27 kb)

Additional file 2: Sample selection. (DOCX 27 kb)

Additional file 3: Statistical analysis: model selection. (DOCX 28 kb)

Additional file 4: Descriptive statistics of healthcare spending. A. General population sample. B. Diabetes sample. C. Depression sample. (DOCX 38 kb)

Additional file 5: Complete LMM model estimates for the general population. (DOCX 36 kb)

## Abbreviations

AIC: Akaike Information Criteria
BIC: Bayesian Information Criterion
CoV: Coefficient of Variation
DCG: Diagnosis-based Cost Group
DFHCS: Dutch Diabetes Federation Health Care Standard
DPHM: Dutch Public Health Monitor
DTG: Diagnosis Treatment Group
GP: General Practitioner
IGZ: Health Care Inspectorate
LMM: Linear Mixed Models
LRT: Likelihood Ratio Test
NIVEL: Netherlands Institute for Health Services Research
PC: Per Capita
PCG: Pharmacy-based Cost Group
PHM: Population Health Management
RIVM: National Institute of Public Health and the Environment
