# Supplementary material for: Unraveling the drivers of regional variation in healthcare spending by analyzing prevalent chronic diseases
Source: BMC Health Serv Res. 2018 May 3;18:323. doi: 10.1186/s12913-018-3128-4 (PMC5934839; doi:10.1186/s12913-018-3128-4)
Supplement: Supplementary file 1 — Data and sources. (DOCX 27 kb) [file 12913_2018_3128_MOESM1_ESM.docx]

**Additional file 1: DATA AND SOURCES**

|  | **Variable** | **Operationalization** | | **Type** | **Data source** |
| --- | --- | --- | --- | --- | --- |
|  |  |  |  |  |  |
| **Supply** | Provider concentration | Number of providers per 1000 population | | ratio | Netherlands Institute for Health Services Research (NIVEL), the National Institute of Public Health and the Environment (RIVM) and the Health Care Inspectorate (IGZ). |
|  |  |  | GP |  |  |
|  |  |  | Pharmacy |  |  |
|  |  |  | Hospital |  |  |
|  |  |  | Physiotherapist |  |  |
| **Demographics** | Age | Age in years | | ratio | Statistics Netherlands |
|  |  |  | |  |  |
|  | Gender | Percentage female | |  | Statistics Netherlands |
| **Health status** | Self-reported health status | How do you perceive your health in general? | | categorical (good - fair - poor) | Dutch Health Monitor survey 2012 |
|  | Claims data health status | Number of Pharmacy Cost Groups in 2008 | | ratio | Vektis 2008/2012 |
|  |  | Number of Pharmacy Cost Groups in 2012 | | ratio |  |
|  |  | Type of Diagnosis-based Cost Group in 2008 | | categorical (none, 1-13) |  |
|  |  | Type of Diagnosis-based Cost Group in 2012 | | categorical (none, 1-15) |  |
|  |  | Number of chronic illnesses | | categorical (none, 1-3) | Claims data Vektis 2012 |
| **Supply** | Accessibility | Distance to nearest provider in meters | | ratio | Statistics Netherlands |
|  |  |  | GP |  |  |
|  |  |  | Pharmacy |  |  |
|  |  |  | Hospital |  |  |
|  |  |  | Physiotherapist |  |  |
| **Outcome variable** | Healthcare spending | Natural log of euro's spend on healthcare 2012 | | ratio | Claims data Vektis 2012 |
|  |  |  | Total curative healthcare spending |  |  |
|  |  |  | GP |  |  |
|  |  |  | Pharmacy |  |  |
|  |  |  | Specialized care |  |  |
|  |  |  | Physiotherapist |  |  |
|  |  |  | Mental health care |  |  |
|  |  |  |  |  |  |

*^GP: General Practitioner^*
