# Supplementary material for: Unraveling the drivers of regional variation in healthcare spending by analyzing prevalent chronic diseases
Source: BMC Health Serv Res. 2018 May 3;18:323. doi: 10.1186/s12913-018-3128-4 (PMC5934839; doi:10.1186/s12913-018-3128-4)
Supplement: Supplementary file 2 — Sample selection. (DOCX 27 kb) [file 12913_2018_3128_MOESM2_ESM.docx]

**Additional file 2: SAMPLE SELECTION**

| Sample: | **General population** | | **Diabetes** |  | **Depression** | |
| --- | --- | --- | --- | --- | --- | --- |
| *Reason* | *included* | *excluded* | *included* | *excluded* | *included* | *excluded* |
| Participants of the Dutch Health Monitor survey | 387195 |  |  |  |  |  |
| Link available with claims data | 362905 | 24290 |  |  |  |  |
| Who are inhabitants of one of the 18 PHM sites | 138732 | 224173 |  |  |  |  |
| Without missings in explanatory variables | 56277 | 82455 |  |  |  |  |
| Without missings in dependent variable | 45979 | 10298 |  |  |  |  |
| Without 1% outliers in dependent variable (at the top) | 44695 | 1284 |  |  |  |  |
| Without negative values for the dependent variable | 44694 | 1 |  |  |  |  |
|  |  |  |  |  |  |  |
| **Study sample total population** | **44694** |  | 44694 |  | 44694 |  |
| **Study sample diabetes** |  |  | **10767** | 33927 |  |  |
| **Study sample depression** |  |  |  |  | **3735** | 40959 |

*^PHM: Population Health Management^*
