# Supplementary material for: Unraveling the drivers of regional variation in healthcare spending by analyzing prevalent chronic diseases
Source: BMC Health Serv Res. 2018 May 3;18:323. doi: 10.1186/s12913-018-3128-4 (PMC5934839; doi:10.1186/s12913-018-3128-4)
Supplement: Supplementary file 3 — Statistical analysis: model selection. (DOCX 28 kb) [file 12913_2018_3128_MOESM3_ESM.docx]

**Additional file 3: STATISTICAL ANALYSIS: MODEL SELECTION**

The selection of a method for the analysis of healthcare spending data is not straightforward, as difficulties arise due the distribution of healthcare spending, which is known to be non-negative, skewed and heavy-tailed. However, in general, generalized linear models (GLM) are used as it deals with the skewed distribution of healthcare spending [1, 2]. GLM requires a mean function and a variance function to be specified beforehand based on the probability distribution of the dependent variable. The mean function relates the mean to some function of covariates and the variance function relates the mean to the variance on the raw scale. For spending data, options for distributions typically include (Generalized) Gamma, Poisson, inverse Gaussian and Gaussian distributions. Link functions include identity, inverse square or power. A major advantage of GLM over Ordinary Least Squares (OLS) using a log-transformed response is that the expected value of the response on the original response scale can be retrieved straightforwardly, without the need for a specific retransformation.

As the gamma family and log link is often used in previous literature for handling data on healthcare spending (e.g. [3, 4]), we used this distribution and link as a starting point. For the gamma distribution the variance is a square function of the mean, which can be checked by performing the modified Park test [2]. We performed the modified Park test by fitting the GLMM for the log of the squared residuals using the log of the predicted y. The coefficient of the log of the predicted y indicates the functional form of the underlying distribution: beta ≈ 0 indicates Gaussian distribution; beta ≈ 1 Poisson; beta ≈ 2 gamma; and beta ≈ 3 indicates inverse Gaussian or Wald distribution. However, due to computational problems, STATA was not able to fit the GLMM with gamma family and log link for the large number of observations and variables in addition to the nested structure. However, in much smaller samples the model with gamma family and log link did converge. Therefore, we consecutively tried fitting Poisson, inverse Gaussian and Gaussian distributions. Due to aforementioned computational problems, STATA was only able to fit the model using the Gaussian distribution. These sorts of computational problems are seen before in e.g. Tsiachristas and Rutten-van Mölken [5] and Mohnen, Molema [4]. In order to refrain from losing too much statistical power we chose a different model instead; he only link option in combination with Gaussian distribution is identity (i.e. Linear Mixed Models (LMM))

A disadvantage of using the Gaussian distribution is that the results are sensitive to extreme values, can produce out-of-range values as negative predicted spending, and is likely to be inefficient for small to medium sample sizes since the underlying distribution in reality is not normal [6]. To accommodate a Gaussian distribution, we decided to log-transform the outcome variable. Modeling the log-transformed variable instead of the raw cost variable may solve the problem of skewness and allows for comparison on the log-transformed scale. Although the results are more precise and robust by the transformation, a major drawback is that the interpretation of the results are not straightforward. The comparison of the means of the log-transformed scale cannot be translated directly to comparison of means on the raw scale by inverting the log, as the log of the expected value does not equal the expected value of the log (i.e. ln(E[y|x]) ≠ E[ln(y|x)]). Appropriate solutions for this problem (e.g. Duan’s non-parametric smearing factor [7]) may be applied to transform the contribution of the covariates. However, variance estimates remain on the log-scale. As the results of this study focuses on the variances and therefore still had to be displayed on the log-scale, we decided not to retransform the covariates.

References

1. Manning, W.G., A. Basu, and J. Mullahy, *Generalized modeling approaches to risk adjustment of skewed outcomes data.* J Health Econ, 2005. **24**(3): p. 465-88.

2. Manning, W.G. and J. Mullahy, *Estimating log models: to transform or not to transform?* J Health Econ, 2001. **20**(4): p. 461-94.

3. Iversen, T., et al., *Comparative Analysis of Treatment Costs in EUROHOPE.* Health Economics, 2015. **24**: p. 5-22.

4. Mohnen, S.M., et al., *Cost Variation in Diabetes Care across Dutch Care Groups?* Health Serv Res, 2016.

5. Tsiachristas, A. and M. Rutten-van Mölken, *Exploring the variability of patient costs in disease management programs: A hierarchical modelling approach.* Applied Economics, 2014. **46**(9): p. 940-951.

6. Mihaylova, B., et al., *Review of Statistical Methods for Analysing Healthcare Resources and Costs.* Health Economics, 2011. **20**(8): p. 897-916.

7. Duan, N., *Smearing Estimate: A Nonparametric Retransformation Method.* Journal of the American Statistical Association, 1983. **78**(383): p. 605-610.
