# Supplementary material for: Unraveling the drivers of regional variation in healthcare spending by analyzing prevalent chronic diseases
Source: BMC Health Serv Res. 2018 May 3;18:323. doi: 10.1186/s12913-018-3128-4 (PMC5934839; doi:10.1186/s12913-018-3128-4)
Supplement: Supplementary file 4 — Descriptive statistics of healthcare spending. A. General population sample. B. Diabetes sample. C. Depression sample. (DOCX 38 kb) [file 12913_2018_3128_MOESM4_ESM.docx]

**Additional file 4: DESCRIPTIVE STATISTICS OF HEALTHCARE SPENDING PER REGION**

| 1. **General population sample** | | |  |  |  |  |  |  |  |  |  |  |
| --- | --- | --- | --- | --- | --- | --- | --- | --- | --- | --- | --- | --- |
|  |  |  |  |  |  |  |  |  |  |  |  |  |
| **region** | **n** |  | **total** |  | **gp** |  | **hospital** |  | **pharmacy** | | **mental healthcare** | |
|  | reality | sample | mean | sd | mean | sd | mean | sd | mean | sd | mean | sd |
| 1 | 170247 | 2898 | 3886 | 4319 | 215 | 145 | 1955 | 3395 | 878 | 1042 | 151 | 966 |
| 2 | 648250 | 1967 | 3277 | 4269 | 202 | 140 | 1892 | 3436 | 628 | 799 | 93 | 995 |
| 3 | 104883 | 786 | 4024 | 5132 | 189 | 122 | 2129 | 3849 | 796 | 1246 | 87 | 981 |
| 4 | 269786 | 4032 | 4366 | 4994 | 203 | 127 | 2300 | 3984 | 886 | 1055 | 107 | 924 |
| 5 | 182888 | 660 | 3845 | 5110 | 216 | 166 | 2217 | 3989 | 771 | 1298 | 105 | 771 |
| 6 | 418896 | 977 | 3315 | 4494 | 175 | 123 | 1797 | 3405 | 750 | 1689 | 119 | 867 |
| 7 | 274957 | 2003 | 3655 | 4874 | 195 | 131 | 2219 | 4076 | 684 | 990 | 110 | 903 |
| 8 | 518308 | 6975 | 4104 | 4905 | 194 | 144 | 2292 | 3948 | 786 | 1081 | 104 | 974 |
| 9 | 113025 | 238 | 3473 | 4803 | 174 | 91 | 1873 | 3586 | 830 | 1551 | 160 | 1170 |
| 10 | 183053 | 2256 | 4413 | 5007 | 221 | 140 | 2307 | 3756 | 905 | 1085 | 170 | 1459 |
| 11 | 87667 | 779 | 4316 | 5077 | 197 | 125 | 2551 | 4172 | 896 | 1331 | 130 | 1078 |
| 12 | 687486 | 7470 | 3688 | 4553 | 185 | 118 | 1979 | 3655 | 790 | 1049 | 67 | 837 |
| 13 | 126698 | 2601 | 4163 | 5106 | 210 | 144 | 2433 | 4165 | 806 | 1116 | 76 | 762 |
| 14 | 60680 | 299 | 3491 | 4513 | 198 | 123 | 1979 | 3817 | 707 | 1159 | 64 | 400 |
| 15 | 40529 | 88 | 3131 | 4117 | 159 | 95 | 1696 | 2905 | 750 | 1591 | 14 | 70 |
| 16 | 293083 | 4231 | 3936 | 4823 | 201 | 127 | 2323 | 3968 | 753 | 1077 | 89 | 706 |
| 17 | 13692 | 348 | 4172 | 4750 | 225 | 137 | 2251 | 3803 | 891 | 918 | 45 | 360 |
| 18 | 1034788 | 6086 | 4180 | 4976 | 201 | 122 | 2448 | 4087 | 860 | 1164 | 103 | 896 |
| total | 5228916 | 44694 | 3973 | 4816 | 199 | 132 | 2215 | 3870 | 806 | 1104 | 101 | 923 |
|  |  |  |  |  |  |  |  |  |  |  |  |  |
| **B. Diabetes sample** | |  |  |  |  |  |  |  |  |  |  |  |
|  |  |  |  |  |  |  |  |  |  |  |  |  |
| **region** | **n** |  | **total** |  | **gp** |  | **hospital** |  | **pharmacy** | | **mental healthcare** | |
|  | reality | sample | mean | sd | mean | sd | mean | sd | mean | sd | mean | sd |
| 1 |  | 687 | 4723 | 4298 | 240 | 152 | 2074 | 3351 | 1181 | 1002 | 125 | 800 |
| 2 |  | 410 | 4135 | 4574 | 220 | 145 | 2022 | 3527 | 964 | 1214 | 16 | 217 |
| 3 |  | 187 | 4814 | 4873 | 184 | 106 | 2485 | 3665 | 974 | 902 | 16 | 114 |
| 4 |  | 1104 | 5277 | 5348 | 217 | 130 | 2515 | 4208 | 1128 | 927 | 104 | 1163 |
| 5 |  | 95 | 4591 | 4616 | 234 | 128 | 2143 | 3004 | 1086 | 1283 | 165 | 1145 |
| 6 |  | 185 | 3884 | 4238 | 187 | 131 | 1804 | 3180 | 870 | 984 | 129 | 836 |
| 7 |  | 393 | 4448 | 4765 | 209 | 154 | 2292 | 3883 | 908 | 942 | 147 | 1468 |
| 8 |  | 1632 | 5010 | 5208 | 209 | 151 | 2598 | 4172 | 1020 | 956 | 76 | 726 |
| 9 |  | 45 | 3691 | 3022 | 166 | 71 | 1357 | 1767 | 1333 | 1955 | 20 | 135 |
| 10 |  | 550 | 4886 | 4859 | 235 | 135 | 2262 | 3620 | 1272 | 1445 | 63 | 723 |
| 11 |  | 231 | 4949 | 5275 | 212 | 146 | 2541 | 4091 | 1095 | 1089 | 189 | 1564 |
| 12 |  | 1907 | 4369 | 4558 | 197 | 126 | 2044 | 3555 | 1052 | 1197 | 35 | 523 |
| 13 |  | 589 | 5244 | 5276 | 237 | 174 | 2725 | 4271 | 1108 | 1175 | 88 | 1242 |
| 14 |  | 73 | 4114 | 4824 | 227 | 139 | 2039 | 4230 | 802 | 716 | 134 | 689 |
| 15 |  | 15 | 3493 | 3467 | 177 | 147 | 1417 | 2957 | 789 | 639 | 0 | 0 |
| 16 |  | 940 | 4586 | 4680 | 209 | 138 | 2343 | 3702 | 1002 | 1212 | 84 | 767 |
| 17 |  | 75 | 4695 | 4345 | 242 | 159 | 2150 | 3083 | 1052 | 770 | 7 | 41 |
| 18 |  | 1649 | 5067 | 5274 | 215 | 131 | 2802 | 4350 | 1103 | 1019 | 83 | 626 |
| total |  | 10767 | 4786 | 4938 | 214 | 140 | 2389 | 3914 | 1068 | 1097 | 79 | 831 |
|  |  |  |  |  |  |  |  |  |  |  |  |  |
| **C. Depression sample** | |  |  |  |  |  |  |  |  |  |  |  |
|  |  |  |  |  |  |  |  |  |  |  |  |  |
| **region** | **n** |  | **total** |  | **gp** |  | **hospital** |  | **pharmacy** | | **mental healthcare** | |
|  | reality | sample | mean | sd | mean | sd | mean | sd | mean | sd | mean | sd |
| 1 |  | 300 | 4491 | 4590 | 219 | 142 | 1936 | 3298 | 896 | 906 | 657 | 1859 |
| 2 |  | 184 | 3415 | 4812 | 199 | 126 | 1705 | 3576 | 629 | 718 | 456 | 2203 |
| 3 |  | 75 | 4530 | 5520 | 179 | 106 | 2484 | 4581 | 711 | 774 | 356 | 1767 |
| 4 |  | 330 | 4960 | 5261 | 216 | 156 | 2229 | 3660 | 987 | 1013 | 628 | 2092 |
| 5 |  | 61 | 4383 | 5546 | 245 | 189 | 2308 | 4222 | 802 | 858 | 596 | 1836 |
| 6 |  | 97 | 3366 | 4110 | 208 | 134 | 1599 | 3003 | 690 | 1015 | 458 | 1605 |
| 7 |  | 138 | 3804 | 5147 | 216 | 182 | 1685 | 3209 | 802 | 983 | 654 | 2591 |
| 8 |  | 502 | 4971 | 5633 | 209 | 159 | 2404 | 4007 | 864 | 919 | 752 | 2815 |
| 9 |  | 37 | 2950 | 4543 | 146 | 52 | 1544 | 3563 | 541 | 847 | 407 | 1301 |
| 10 |  | 203 | 5034 | 6419 | 222 | 143 | 1897 | 3730 | 989 | 1082 | 1005 | 3734 |
| 11 |  | 69 | 5459 | 5514 | 239 | 177 | 2524 | 3492 | 1259 | 1896 | 630 | 2102 |
| 12 |  | 647 | 4032 | 4857 | 195 | 120 | 1779 | 3139 | 892 | 1176 | 451 | 2210 |
| 13 |  | 212 | 4823 | 5349 | 232 | 148 | 2548 | 4432 | 902 | 959 | 330 | 1021 |
| 14 |  | 24 | 3474 | 4530 | 181 | 67 | 1251 | 1687 | 1314 | 3191 | 198 | 555 |
| 15 |  | 12 | 2941 | 4538 | 161 | 99 | 1758 | 3651 | 433 | 479 | 101 | 172 |
| 16 |  | 402 | 4746 | 5515 | 220 | 150 | 2553 | 4416 | 814 | 1307 | 550 | 1680 |
| 17 |  | 15 | 5799 | 3699 | 306 | 208 | 2715 | 3347 | 1147 | 821 | 856 | 1503 |
| 18 |  | 427 | 4592 | 5031 | 212 | 134 | 2249 | 3747 | 958 | 1319 | 623 | 1932 |
| total |  | 3735 | 4493 | 5233 | 211 | 144 | 2125 | 3736 | 881 | 1126 | 588 | 2216 |

*^GP: general practicioner; sd: standard deviatio^*
