# Supplementary material for: Unraveling the drivers of regional variation in healthcare spending by analyzing prevalent chronic diseases
Source: BMC Health Serv Res. 2018 May 3;18:323. doi: 10.1186/s12913-018-3128-4 (PMC5934839; doi:10.1186/s12913-018-3128-4)
Supplement: Supplementary file 5 — Complete LMM model estimates for the general population. (DOCX 36 kb) [file 12913_2018_3128_MOESM5_ESM.docx]

**Additional file 5: COMPLETE LMM MODEL ESTIMATES FOR THE GENERAL POPULATION**

|  |  |  |  |  | Model 0 | | | Model 1 | | | Model 2 | | | Model 3 | | | Model 4 | | | Model 5 | | | Model 6 | | | Model 7 | | | Model 8 | | | Model 9 | | | Model 10 | | |
| --- | --- | --- | --- | --- | --- | --- | --- | --- | --- | --- | --- | --- | --- | --- | --- | --- | --- | --- | --- | --- | --- | --- | --- | --- | --- | --- | --- | --- | --- | --- | --- | --- | --- | --- | --- | --- | --- |
|  | **Variable** | |  |  | **B (se)** | | | **B (se)** | | | **B (se)** | | | **B (se)** | | | **B (se)** | | | **B (se)** | | | **B (se)** | | | **B (se)** | | | **B (se)** | | | **B (se)** | | | **B (se)** | | |
| **fixed effects** | | intercept (patient level) | |  | 7,64 (0,03)*** | | | 7 (0,04)*** | | | 6,37 (0,03)*** | | | 6,30 (0,04)*** | | | 6,18 (0,03)*** | | | 6,18 (0,03)*** | | | 6,20 (0,03)*** | | | 6,19 (0,03)*** | | | 6,20 (0,03)*** | | | 6,21 (0,03)*** | | | 6,21 (0,03)*** | | |
| demand | | pension age | |  |  |  |  | 0,02 (0,00)*** | | | 0,01 (0,00)*** | | | 0,01 (0,00)*** | | | 0,01 (0,00)*** | | | 0,01 (0,00)*** | | | 0,01 (0,00)*** | | | 0,01 (0,00)*** | | | 0,01 (0,00)*** | | | 0,01 (0,00)*** | | | 0,01 (0,00)*** | | |
|  |  | gender | |  |  |  |  | -0,02 (0,01)* | | | -0,05 (0,01)*** | | | 0,02 (0,01) | | | 0,06 (0,01)*** | | | 0,04 (0,01)*** | | | 0,03 (0,01)*** | | | 0,03 (0,01)*** | | | 0,03 (0,01)*** | | | 0,03 (0,01)*** | | | 0,03 (0,01)*** | | |
|  | self-reported health status | | |  |  |  |  |  |  |  |  | | |  | | |  | | |  | | |  | | |  | | |  | | |  | | |  | | |
|  |  | fair |  |  |  |  |  |  |  |  | 0,71 (0,01)*** | | |  | | |  | | | 0,44 (0,01)*** | | | 0,44 (0,01)*** | | | 0,44 (0,01)*** | | | 0,44 (0,01)*** | | | 0,43 (0,01)*** | | | 0,44 (0,01)*** | | |
|  |  | poor |  |  |  |  |  |  |  |  | 1,30 (0,02)*** | | |  | | |  | | | 0,77 (0,02)*** | | | 0,77 (0,02)*** | | | 0,77 (0,02)*** | | | 0,77 (0,02)*** | | | 0,77 (0,02)*** | | | 0,77 (0,02)*** | | |
|  | claims data derived health status | | DCG * | 1 |  |  |  |  |  |  |  |  |  | 0,45 (0,03)*** | | | 1,00 (0,03)*** | | | 0,90 (0,03)*** | | | 0,90 (0,03)*** | | | 0,90 (0,03)*** | | | 0,90 (0,03)*** | | | 0,90 (0,03)*** | | | 0,90 (0,03)*** | | |
|  |  |  |  | 2 |  |  |  |  |  |  |  |  |  | 0,28 (0,04)*** | | | 0,89 (0,02)*** | | | 0,79 (0,02)*** | | | 0,79 (0,02)*** | | | 0,79 (0,02)*** | | | 0,79 (0,02)*** | | | 0,79 (0,02)*** | | | 0,80 (0,02)*** | | |
|  |  |  |  | 3 |  |  |  |  |  |  |  |  |  | 0,34 (0,05)*** | | | 0,90 (0,02)*** | | | 0,81 (0,02)*** | | | 0,81 (0,02)*** | | | 0,81 (0,02)*** | | | 0,81 (0,02)*** | | | 0,81 (0,02)*** | | | 0,81 (0,02)*** | | |
|  |  |  |  | 4 |  |  |  |  |  |  |  |  |  | 0,56 (0,05)*** | | | 1,11 (0,02)*** | | | 1,02 (0,02)*** | | | 1,02 (0,02)*** | | | 1,02 (0,02)*** | | | 1,02 (0,02)*** | | | 1,02 (0,02)*** | | | 1,02 (0,02)*** | | |
|  |  |  |  | 5 |  |  |  |  |  |  |  |  |  | 0,42 (0,05)*** | | | 1,21 (0,02)*** | | | 1,09 (0,02)*** | | | 1,09 (0,02)*** | | | 1,09 (0,02)*** | | | 1,09 (0,02)*** | | | 1,09 (0,02)*** | | | 1,09 (0,02)*** | | |
|  |  |  |  | 6 |  |  |  |  |  |  |  |  |  | 0,58 (0,06)*** | | | 1,19 (0,02)*** | | | 1,04 (0,02)*** | | | 1,04 (0,02)*** | | | 1,04 (0,02)*** | | | 1,04 (0,02)*** | | | 1,04 (0,02)*** | | | 1,04 (0,02)*** | | |
|  |  |  |  | 7 |  |  |  |  |  |  |  |  |  | 0,95 (0,08)*** | | | 1,28 (0,03)*** | | | 1,13 (0,03)*** | | | 1,13 (0,03)*** | | | 1,13 (0,03)*** | | | 1,13 (0,03)*** | | | 1,13 (0,03)*** | | | 1,13 (0,03)*** | | |
|  |  |  |  | 8 |  |  |  |  |  |  |  |  |  | 1,05 (0,09)*** | | | 1,50 (0,06)*** | | | 1,30 (0,06)*** | | | 1,30 (0,06)*** | | | 1,30 (0,06)*** | | | 1,30 (0,06)*** | | | 1,30 (0,06)*** | | | 1,30 (0,06)*** | | |
|  |  |  |  | 9 |  |  |  |  |  |  |  |  |  | 0,95 (0,09)*** | | | 1,41 (0,04)*** | | | 1,25 (0,04)*** | | | 1,25 (0,04)*** | | | 1,25 (0,04)*** | | | 1,25 (0,04)*** | | | 1,25 (0,04)*** | | | 1,25 (0,04)*** | | |
|  |  |  |  | 10 |  |  |  |  |  |  |  |  |  | 0,72 (0,12)*** | | | 1,54 (0,04)*** | | | 1,38 (0,04)*** | | | 1,38 (0,04)*** | | | 1,38 (0,04)*** | | | 1,38 (0,04)*** | | | 1,38 (0,04)*** | | | 1,38 (0,04)*** | | |
|  |  |  |  | 11 |  |  |  |  |  |  |  |  |  | 0,99 (0,19)*** | | | 1,70 (0,12)*** | | | 1,45 (0,12)*** | | | 1,45 (0,12)*** | | | 1,45 (0,12)*** | | | 1,45 (0,12)*** | | | 1,45 (0,12)*** | | | 1,45 (0,12)*** | | |
|  |  |  |  | 12 |  |  |  |  |  |  |  |  |  | 0,89 (0,08)*** | | | 1,33 (0,06)*** | | | 1,14 (0,06)*** | | | 1,14 (0,06)*** | | | 1,14 (0,06)*** | | | 1,14 (0,06)*** | | | 1,14 (0,06)*** | | | 1,14 (0,06)*** | | |
|  |  |  |  | 13 |  |  |  |  |  |  |  |  |  | 0,94 (0,14)*** | | | 2,06 (0,10)*** | | | 1,83 (0,09)*** | | | 1,83 (0,09)*** | | | 1,83 (0,09)*** | | | 1,83 (0,09)*** | | | 1,82 (0,09)*** | | | 1,83 (0,09)*** | | |
|  |  |  |  | 14 |  |  |  |  |  |  |  |  |  |  |  |  | 2,05 (0,32)*** | | | 1,96 (0,31)*** | | | 1,96 (0,31)*** | | | 1,96 (0,31)*** | | | 1,96 (0,31)*** | | | 1,96 (0,31)*** | | | 1,96 (0,30)*** | | |
|  |  |  |  | 15 |  |  |  |  |  |  |  |  |  |  |  |  | 2,28 (0,52)*** | | | 2,00 (0,50)*** | | | 2,00 (0,50)*** | | | 2,00 (0,50)*** | | | 1,99 (0,50)*** | | | 2,00 (0,50)*** | | | 1,99 (0,50)*** | | |
|  |  |  | PCG 2008 |  |  |  |  |  |  |  |  |  |  | 0,33 (0,01)*** | | |  |  |  |  |  |  |  |  |  |  |  |  |  |  |  |  |  |  |  |  |  |
|  |  |  | PCG 2012 |  |  |  |  |  |  |  |  |  |  |  |  |  | 0,32 (0,01)*** | | | 0,25 (0,01)*** | | | 0,25 (0,01)*** | | | 0,25 (0,01)*** | | | 0,25 (0,01)*** | | | 0,25 (0,01)*** | | | 0,25 (0,01)*** | | |
| supply | | | | |  |  |  |  |  |  |  |  |  |  |  |  |  |  |  |  |  |  |  |  |  |  |  |  |  |  |  |  |  |  |  |  |  |
|  | distance to care provider in meters | | | |  |  |  |  |  |  |  |  |  |  |  |  |  |  |  |  |  |  |  |  |  |  |  |  |  |  |  |  |  |  |  |  |  |
|  |  | GP | | |  |  |  |  |  |  |  |  |  |  |  |  |  |  |  |  |  |  | 0,00 (0,00)*** | | |  |  |  |  |  |  |  |  |  | 0,00 (0,00)*** | | |
|  |  | pharmacy | | |  |  |  |  |  |  |  |  |  |  |  |  |  |  |  |  |  |  |  |  |  | -0,00 (0,00)*** | | |  |  |  |  |  |  | 0,00 (0,00)*** | | |
|  |  | hospital | | |  |  |  |  |  |  |  |  |  |  |  |  |  |  |  |  |  |  |  |  |  |  |  |  | 0,00 (0,00)*** | | |  |  |  | 0,00 (0,00)*** | | |
|  |  | physical therapist | | |  |  |  |  |  |  |  |  |  |  |  |  |  |  |  |  |  |  |  |  |  |  |  |  |  |  |  | 0,00 (0,00)*** | | | 0,00 (0,00)*** | | |
| **Random effects** | | |  |  |  | | |  | | |  | | |  | | |  | | |  | | |  | | |  | | |  | | |  | | |  | | |
|  | intercept – variance at the region level | |  |  | 0,02 (0,01) | | | 0,01 (0,00) | | | 0,00 (0,00) | | | 0,01 (0,00) | | | 0,01 (0,00) | | | 0,00 (0,00) | | | 0,00 (0,00) | | | 0,00 (0,00) | | | 0,00 (0,00) | | | 0,00 (0,00) | | | 0,00 (0,00) | | |
|  | random error – variance at the individual level | |  |  | 1,24 (0,01) | | | 1,20 (0,01) | | | 1,02 (0,01) | | | 1,10 (0,01) | | | 0,81 (0,01) | | | 0,75 (0,01) | | | 0,75 (0,01) | | | 0,75 (0,01) | | | 0,75 (0,01) | | | 0,75 (0,01) | | | 0,75 (0,01) | | |
| **Post estimation statistics** | | |  |  |  | | |  | | |  | | |  | | |  | | |  | | |  | | |  | | |  | | |  | | |  | | |
|  | ICC | |  |  | 0,02 (0,01) | | | 0,01 (0,00) | | | 0,00 (0,00) | | | 0,01 (0,00) | | | 0,01 (0,00) | | | 0,01 (0,00) | | | 0,01 (0,00) | | | 0,01 (0,00) | | | 0,01 (0,00) | | | 0,01 (0,00) | | | 0,00 (0,00) | | |
|  | AIC | |  |  | 136507 | | | 135161 | | | 127804 | | | 131208 | | | 117692 | | | 114311 | | | 114305 | | | 114310 | | | 114308 | | | 114293 | | | 114297 | | |
|  | BIC | |  |  | 136533 | | | 135205 | | | 127865 | | | 131373 | | | 117875 | | | 114512 | | | 114514 | | | 114519 | | | 114517 | | | 114502 | | | 114532 | | |
|  | log likelihood | |  |  | -68250 | | | -67576 | | | -63895 | | | -65585 | | | -58825 | | | -57133 | | | -57129 | | | -57131 | | | -57130 | | | -57123 | | | -57122 | | |
|  | likelihood ratio test | |  |  |  | | | 1349*** | | | 7361*** | | | 3981*** | | | 17501*** | | | 20886*** | | | 8*** | | | 3* | | | 4** | | | 20*** | | | 22*** | | |
|  | PC mean and predicted mean | | | |  | | | 0,47*** | | | 0,45*** | | | 0,26*** | | | 0,15*** | | | 0,14*** | | | 0,14*** | | | 0,14*** | | | 0,14*** | | | 0,14*** | | | 0,14*** | | |
|  | Total variance on the log scale | | | | 1,54 | | | 1,45 | | | 1,04 | | | 1,21 | | | 0,66 | | | 0,57 | | | 0,57 | | | 0,57 | | | 0,57 | | | 0,57 | | | 0,57 | | |

*^B: beta; se: standard error; AIC: Aikaike Information Criterion; BIC: Bayesian Information Criterion; PC: Pearson’s Correlation; GP: general practitioner; PCG: Pharmacy-based Cost Group; DCG: Diagnosis Cost Group; *: p-value < 0.10; **: p-value < 0.05; ***: p-value < 0.01; #:DCG in model 3 is for the year 2008. As from model 4 the year^*
